# Apathy as a Risky Neuropsychiatric Syndrome of Progression From Normal Aging to Mild Cognitive Impairment and Dementia: A Systematic Review and Meta-Analysis

**DOI:** 10.3389/fpsyt.2021.792168

**Published:** 2021-12-20

**Authors:** Zili Fan, Luchun Wang, Haifeng Zhang, Xiaozhen Lv, Lihui Tu, Ming Zhang, Ying Zhang, Caihua Yan, Xin Yu, Huali Wang

**Affiliations:** ^1^Beijing Dementia Key Lab, Dementia Care and Research Center, Peking University Institute of Mental Health (Sixth Hospital), Beijing, China; ^2^NHC Key Laboratory of Mental Health, National Clinical Research Center for Mental Disorders, Peking University Sixth Hospital, Peking University, Beijing, China; ^3^Department of Psychiatry, The Third Affiliated Hospital of Sun Yat-sen University, Guangzhou, China; ^4^Department of Psychiatry, The First Hospital of Hebei Medical University, Shijiazhuang, China

**Keywords:** apathy, normal aging, mild cognitive impairment, dementia, neuropsychiatric syndrome

## Abstract

**Background:** Apathy has been suggested as a potential predictor of mild cognitive impairment (MCI) progression to dementia. Whether it might predict the transition from normal cognitive function to cognitive impairment has been less studied. The current study aimed to provide a comprehensive summary of the evidence on the association between apathy and the transition from normal cognitive function to cognitive impairment.

**Methods:** We searched the PubMed, Embase, and Web of Science databases for longitudinal prospective cohort studies that evaluated apathy at baseline in the cognitively normal population and had cognitive impairment as the outcome. Random effects models were used, and heterogeneity was explored with stratification. The stability of the synthesized result was indicated using sensitivity analysis by excluding one study each time and recalculating the overall effect.

**Results:** Ten studies comprising 26,195 participants were included. Apathy status was available for 22,101 participants. Apathy was present in 1,803 of 22,101 participants (8.16%). Follow-up ranged from 1 to 13 years. The combined odds ratio (OR) of cognitive impairment for patients with apathy was 2.07 (95% CI: 1.43–2.99; I^2^ = 86%), and the combined hazard ratio was 2.70 (95% CI: 1.38–5.27; I^2^ = 94%). The OR meta-analyses for different conversion outcomes were MCI (OR = 3.38, 95% CI: 1.57–7.28; I^2^ =71%), cognitive decline (OR = 1.27, 95% CI: 0.81–2.00; I^2^ = 64%) and dementia (OR = 2.12, 95% CI: 1.32–3.41; I^2^ = 86%). Subgroup analysis suggested that the association between apathy and cognitive impairment changed with age, depression adjustments, apathy measurement, and follow-up time.

**Conclusions:** Apathy was associated with a greater than 2-fold increased risk of progression to cognitive impairment in the cognitively normal population. Future interventions targeting apathy management in the general population may reduce the risk of cognitive impairment.

## Introduction

Dementia has been considered a public health priority, and there is growing interest in identifying predictive factors of cognitive impairment. Increasing evidence suggests that neuropsychiatric symptoms (NPS) have prognostic value in predicting accelerated disease progression and functional decline (1, 2). NPS refers to disturbances of behavior-, emotion-, and thought-related neurodegenerative diseases (3). In many individuals, the first symptoms of dementia might be NPS rather than a change in cognitive function. Thus, NPS might identify cognitively healthy persons at risk of dementia for prevention trials (4). As one of the most common NPSs, the prevalence of apathy was 4.8% in the cognitively normal (CN) population (5).

Apathy has been defined as loss of motivation, characterized by diminished goal-oriented behavior and cognition and reduced emotional expression (6). Apathy is known to contribute to caregiver burden significantly and has negative implications for activities of daily living among those with dementia (7, 8). Recently, a meta-analysis reported that apathy was associated with an approximately 2-fold increased risk of dementia among those visiting memory clinics (9). The association between apathy and dementia weakened with increasing cognitive impairment. This meta-analysis mainly included samples with mild cognitive impairment (MCI) rather than the CN population. The apathy that could predict the CN-cognitive impairment transition was not identical to that of the MCI-dementia transition. For example, one study among the general population showed that apathy at baseline did not have value in predicting the progression from CN to dementia (10).

Previous clinical studies of the associations between apathy and cognitive impairment among CN populations are also inconsistent. For example, one study showed that apathy failed to predict MCI in community-dwelling older adults. However, in sensitivity analyses of MCI subtypes, apathy was associated with non-amnestic MCI but not amnestic MCI (11). In another community-based study, apathy was associated with an approximately 2.3-fold increased risk of MCI (12). The variability of results between studies might be partly explained by methodological differences in the apathy measurement, the lack of adjustment for depression, or the follow-up time.

The inconsistency in results regarding apathy as a predictor of cognitive decline among study findings in CN populations hinders progress in research on apathy in dementia. Furthermore, no systematic review or meta-analysis has examined aspects of apathy as predictors of cognitive decline among CN populations. Therefore, the current study aims to provide a comprehensive summary of the evidence on the association between apathy and the transition to cognitive impairment, including MCI and dementia, among those in the CN population and to further explore the subgroup differences via a meta-analysis of prospective longitudinal studies.

## Methods

This meta-analysis is reported in line with the PRISMA statement. The PRISMA checklist is provided in the Supplementary Material 1.

### Search Strategy and Study Selection

We searched for all cohort studies reporting associations between apathy and the incidence of cognitive impairment published in PubMed, Embase, and Web of Science databases from their inception to August 27, 2020. Briefly, the search strategy included the following terms: (apathy or neuropsychiatric symptoms AND (dementia or cognitive impairment or Alzheimer disease) AND (cohort study OR longitudinal study OR risk) using both medical subject headings and abstract searches (Supplementary Table 2). In addition, articles were included if they (1) involved unselected general populations or populations with normal cognitive function at baseline; (2) utilized a longitudinal prospective cohort study design; (3) reported data regarding the association between apathy and incident cognitive impairment; and (4) were peer-reviewed articles.

Studies were excluded if they (a) used a cross-sectional design; (b) were published in an abstract format; (c) included participants with cognitive impairment at baseline, or (d) were not published in English. References of relevant articles and systematic reviews were also searched for additional studies. References were compiled using Endnote x9, with duplicates removed using this software. The references identified from the literature search were screened based on the titles and abstracts by one reviewer (ZF) to identify potentially relevant articles. Duplicate and ineligible studies were removed.

### Data Extraction and Quality Assessment

Two reviewers (ZF and LW) independently extracted the data and evaluated the quality of the included studies. The inclusion or exclusion of studies was decided based on consensus. Any indecision about study inclusion was discussed with a third reviewer (HW). We extracted the following information from each study included in the meta-analysis: sample settings, sample size, average or median follow-up duration, the number of cognitive impairment cases in the apathy and non-apathy groups, percentage of women, age of participants, the instrument used to measure apathy, diagnostic criteria for cognitive impairment, covariates included in the adjusted models, and adjusted odds ratio (OR)/hazard ratio (HR) estimates. We used the Newcastle-Ottawa Scale (NOS) to analyze the risk of study bias (13), a nine-point scale used to evaluate the risk of bias of a given cohort study based on three criteria: population selection, comparability, and outcome. Higher scores on the NOS indicated a lower risk of bias.

### Data Analysis

For the meta-analyses, we considered ORs as the main effect size. Studies reporting HRs were analyzed separately. The HRs adjusted for confounders in the final model were included in the meta-analysis. Studies with two follow-up times were included in the OR meta-analysis twice. The HR meta-analysis did not include studies reporting HRs by separating the association according to whether regional brain glucose hypometabolism was present rather than the whole sample. A random-effects model was used to address both of the study objectives because of heterogeneity between studies. Heterogeneity was assessed using I^2^ statistics.

Subgroup analyses based on various factors were performed to explore their impact on heterogeneity: sample type, age, percent of women in the sample, duration of the follow-up time, apathy measurement, depression adjustment, APOE status adjustment, diagnostic criteria of the outcome, and different conversion outcomes. In addition, sensitivity analysis to inspect the influence of a single study on the overall result was conducted by omitting studies one by one. Finally, publication bias was assessed by funnel plots and Egger's test. All statistical analyses were performed with STATA statistical software 15.1 and Review Manager 5.3.

## Results

### Search Results and Characteristics of the Included Studies

From 5,549 titles and abstracts, ten studies were included in the final synthesis (Figure 1) (1, 9–12, 14–18). Details regarding the study characteristics of the included studies are presented in Table 1. Among the ten studies comprising 26,195 participants, apathy was diagnosed in 1,803 of 22,101 participants (8.16%). The median population sample size was 1,408 (range: 457–12,452). The median duration of follow-up was 4.3 years (range: 1 and 13 years). The median/mean age was 72.8 years (range: 65.56–79.3), excluding one study that did not report the mean or median age (14), and the median percentage of women was 61.6% (range: 47.2–63.7%).

**Table 1:** Characteristics of the studies included in the meta-analysis.

**References**	**Population setting**	**Sample size**	**Average or median follow-up (year)**	**Age, mean (SD) or median (year)**	**Female, No. (%)**	**Apathy measure**	**Outcome**	**Criteria**	**Reported association or cognitive impairment incidence**	**Adjustments**	**NOS**
Clarke et al. (15)	Community-dwelling older adults	1,136	1	65.56 (8.79)	700 (61.6)	GHQ (≥6.5)	Cognitive decline	3-point reduction in MMSE scores between baseline and follow-up	OR: 1.65 (1.06–2.60)	Age, sex, education, race, and depression	5
Clarke et al. (15)	Community-dwelling older adults	1,136	13	65.56 (8.79)	700 (61.6)	GHQ (≥6.5)	Cognitive decline	3-point reduction in MMSE scores between baseline and follow-up	OR: 1.04 (0.76–1.44)	Age, sex, education, race, and depression	6
Krell-Roesch et al. (1)	Community-dwelling older adults	1,363	4.8	71.1	644 (47.2)	NPI-Q	MCI	revised Mayo Clinic criteria	HR: FDG-PET-/apathy+: 6.85 (3.36, 14.0); FDG-PET+/apathy+: 5.26 (2.48, 11.1); To MCI: 18 convertors in 42 with apathy; 155 convertors in 1,209 without apathy	Age, sex, education, and APOE ε4 status, medical comorbidity and antidepressant medication intake	9
Acosta et al. (10)	Community-dwelling older adults	1,355	3	73.2 (0.17)	64.3 (871)	NPI-Q	Dementia	DSM-IV	RR: 1.4 (0.9–2.3); To dementia: 16 convertors in 121 with apathy; 113 convertors in 1234 without apathy	Age, gender, level of educational attainment, and MCI	8
van der Linde et al. (14)	Population in rural and urban areas	457	2	197 (29.4)^#^	348 (54.5)	GMS-AGECAT	Dementia	DSM-III	To dementia: 1 convertor in 40 with apathy; 4 convertors in 417 without apathy	Age, sex, education, social class, MMSE, subjective and objective memory and ADL	8
Liew (16)	Clinical samples	12,452	4.7	72	7938 (63.7)	NPI-Q	Dementia	DSM-IV	To dementia: 68 convertors in 574 with apathy; 656 convertors in 11,878 without apathy	Age, sex, ethnicity, education, APOE e4 status and use of antidepressants	9
Burke et al. (17)	Database of memory clinics	1567	4	71.2 (10.9)	988 (63)	NPI-Q	AD	NINCDS	HR: 9.51 (5.23-17.31); 193 convertors in 297 with apathy; 372 convertors in 1,144 without apathy	Sex, age, race, APOE ε4, Hispanic origin, family history	8
Geda et al. (12)	Community-dwelling older adults	1,408	5	79.3	704 (50.0)	NPI-Q	MCI, aMCI, non-MCI	Mayo Clinic criteria	HR: 2.26 (1.49–3.41); To dementia: 25 convertors in 57 with apathy; 339 convertors in 1,351 without apathy	Age, sex, education and medical comorbidity	9
Van Dalen et al. (9)	Community-dwelling older adults	3,499	6	74.3 (2.5)	1899 (54.3)	GDS-3A	Dementia	DSM-IV	HR: 1.21 (1.06–1.40); isolate apathy: 1.20 (1.00-1.45); To dementia: 56 convertors in 672 with apathy symptom; 176 convertors in 2,755 without apathy	Age, sex, MMSE, disability, and history of stroke or cardiovascular disease	9
Ceïde et al. (11)	Community-dwelling older adults	542	13.6 months	76.0 (6.7)	299 (55.2)	GDS-3A	MCI, aMCI, non-MCI	1.5 SD below the RBANS	HR: 1.64 (0.99–2.71)	Adjusted for age, education, baseline global cognition (RBANS), and depressive symptoms	7
Masters et al. (18)	Clinical samples	2,416	4.3^*^	77.8 (8.9)^*^	749 (61.5)^*^	NPI-Q	AD	CDR	HR: 3.81 (2.79–5.2)	Adjusted for age, sex, education, race, and APO e4 status	9

* *those who developed CDR > 0;*

# *Only a proportion of participants 75 years and older was available. aMCI, amnestic mild cognitive impairment; CDR, Clinical Dementia Rating; GDS-3A, Geriatric Depression Scale 3 Apathy-related subitems; GMS-AGECAT, Geriatric Mental State Automated Geriatric Examination for Computer Assisted Taxonomy; MCI, mild cognitive impairment; MMSE, Mini-Mental State Examination; NPI-Q, Neuropsychiatric Inventory Questionnaire for clinical informants; GHQ, the 20-item General Health Questionnaire*.

**Figure 1:**
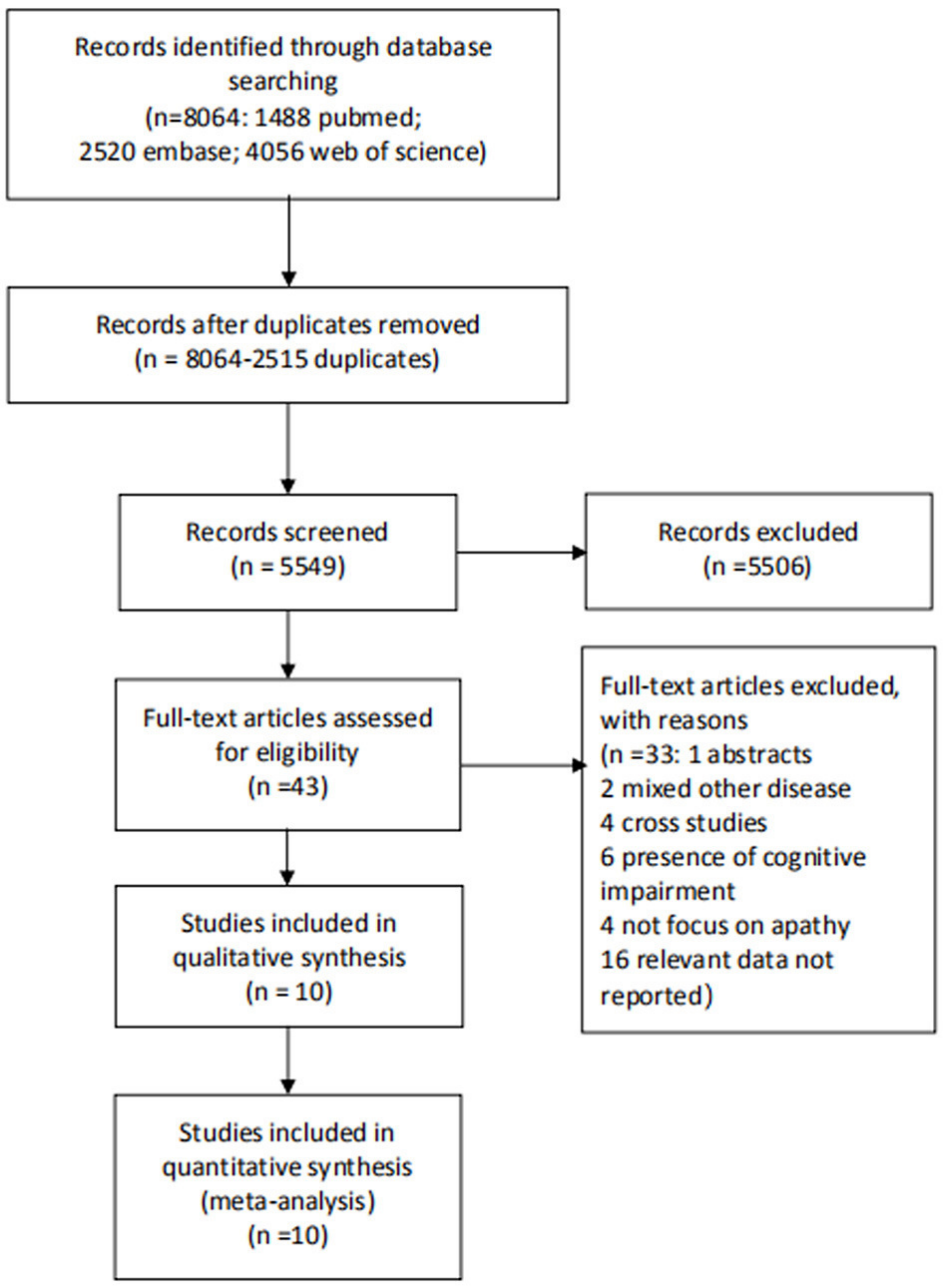
Flow chart of studies selection.

### Risk of Bias Assessment

The risk of bias assessment is shown in Supplementary Figure 1 of the Supplemental documents. Based on total NOS scores, nine studies had a quality score of 7–9, indicating a low risk of bias, and 1 study that included two follow-up times had quality scores of 4–6, indicating a medium risk of bias. The worst scoring categories were outcome assessment, outcome exclusion, and follow-up availability.

### Meta-Analysis of Cognitive Impairment Incidence

**Figure 2:**
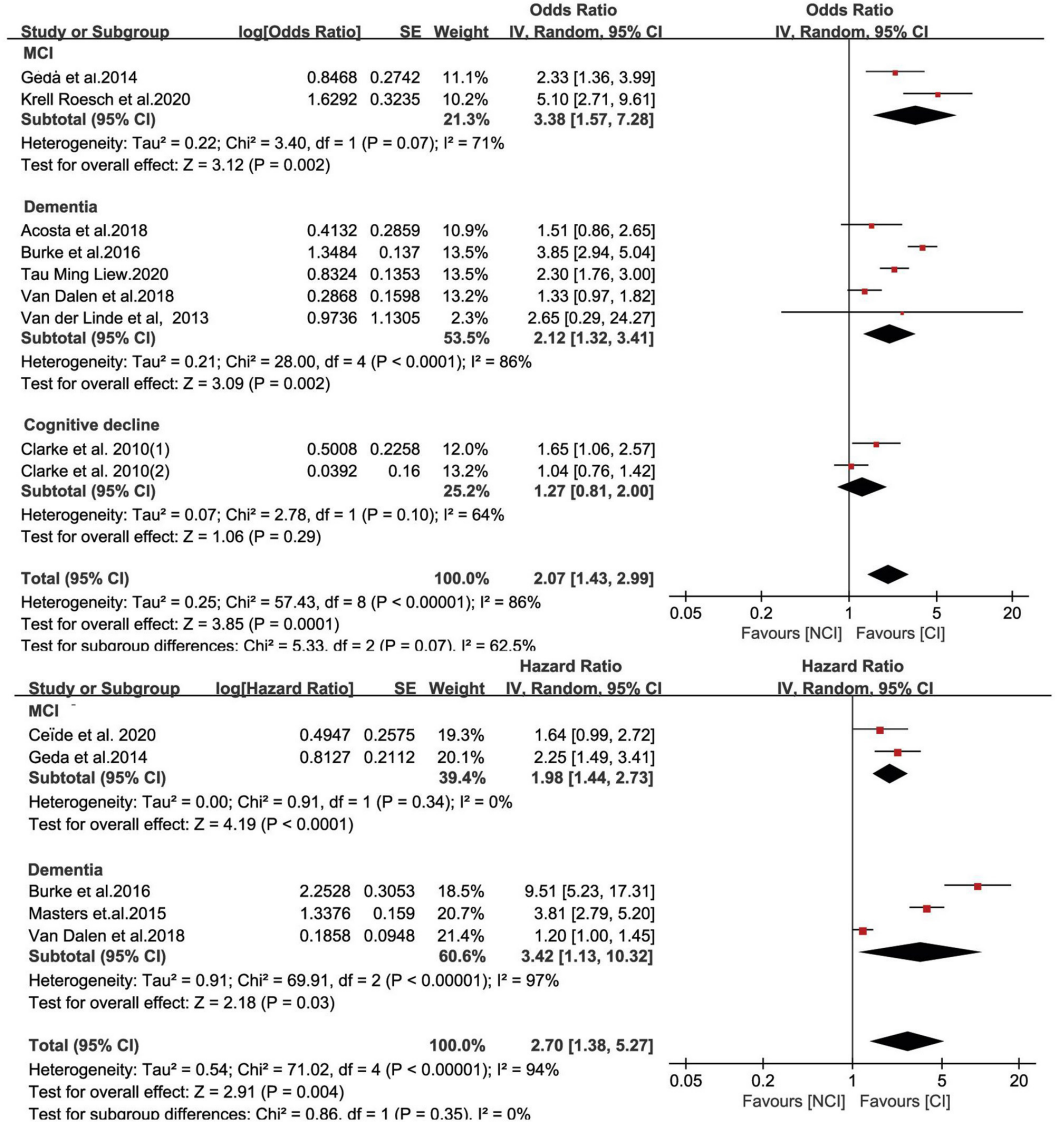
Value of apathy in the prediction of cognitive impairment progression expressed as odds ratios and hazard ratios.

The overall estimate for incident cognitive impairment is shown in Figure 2. The pooled estimate showed that apathetic individuals had a significantly higher risk of developing cognitive impairment than individuals who did not (OR = 2.07, 95% CI: 1.43–2.99). The plot shows high levels of heterogeneity (I^2^ = 86%, *p* <0.001). Pooling the maximally adjusted HR estimates had similar results, with a combined HR of 2.70 (95% CI: 1.38–5.27) and considerable heterogeneity (I^2^ = 94%). After excluding studies one by one from the analysis, the pooled OR slightly changed but remained statistically significant, from 1.85 (95% CI: 1.33–2.57) to 2.29 (95% CI: 1.61–3.25) (Supplementary Figure 2 in the Supplementary documents). The sensitivity analysis for HRs showed similar results, which remained statistically significant, from 2.03 (95% CI: 1.11–3.70) to 3.34 (95% CI: 1.81–6.19) (Supplementary Figure 3 in the supplemental documents). It clearly showed no significant impact of any study on the overall combined results, which remained statistically significant.

### Subgroup Analyses

**Figure 3:**
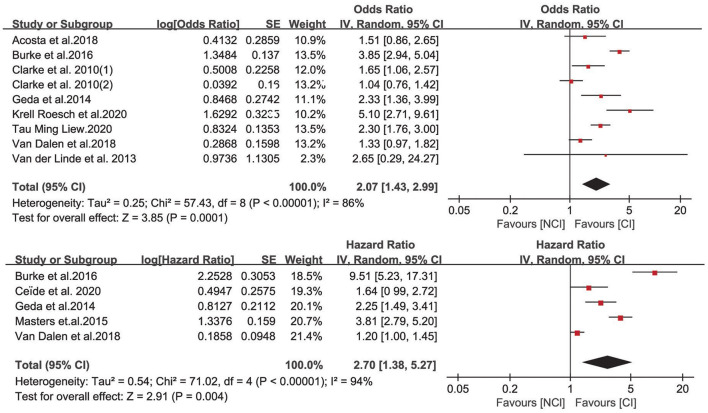
Value of apathy in the prediction of different outcomes expressed as odds ratios and hazard ratios.

**Figure 4:**
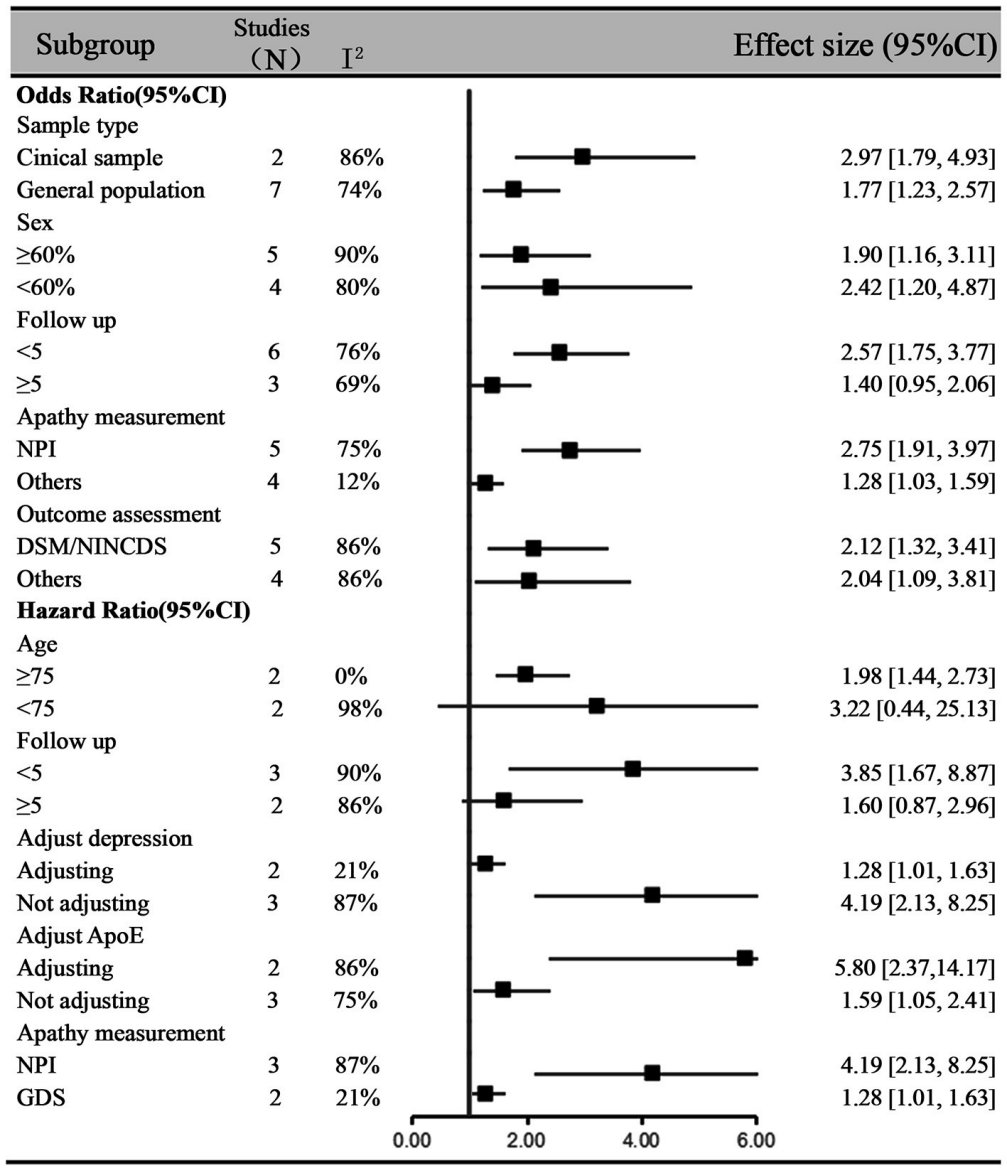
Subgroup analysis of the association between apathy and cognitive impairment based on odds ratios and hazard ratios.

We further performed a meta-analysis on the association between apathy and different conversion outcomes. The pooled estimates showed that apathy could increase the risk of developing MCI (OR = 3.38, 95% CI: 1.57–7.28, I^2^ = 71%) and dementia (OR = 2.12, 95% CI: 1.32–3.41, I^2^ = 86%) (Figure 3). For one study with cognitive decline defined as ≥ 3-point reduction in Mini-Mental State Examination (MMSE) scores, this subgroup did not show significance (OR = 1.27, 95% CI: 0.81–2.00, I^2^ = 64%) (15). For the HR meta-analysis, in the MCI and dementia subgroups, the combined HRs were also significant (HR = 1.98, 95% CI: 1.44–2.73, I^2^ = 0%; HR = 3.42, 95% CI: 1.13–10.32, I^2^ = 97%, respectively). For other subgroup analyses (Figure 4), OR meta-analysis results suggested that the association between apathy and cognitive impairment was enhanced in the clinical sample (OR = 2.97, 95% CI: 1.79–4.93) when the percentage of females was lower than 60% (OR = 2.42, 95% CI: 1.20–4.87) and when neuropsychiatric inventory (NPI) measurements were used (OR = 2.75, 95% CI: 1.91–3.97). The outcome assessment criteria did not result in significant differences. A longer follow-up did not show significance (OR = 1.40, 95% CI: 0.95–2.06). HR subgroup analyses showed similar results. There was no significant difference when longer follow-up times were used (HR = 1.60, 95% CI: 0.87–2.96). There was no significant difference when the age was <75 years (HR = 3.22, 95% CI: 0.44–25.11). ApoE status adjustment did not result in a significantly different outcome. The apathy measurement and depression adjustment subgroups involved the same research, and the use of the Geriatric Depression Scale (GDS) and adjustment for depression showed only a tendency (HR = 1.28, 95% CI: 1.01–1.63, *P* = 0.05).

### Risk of Publication Bias

Egger's tests for the OR and HR meta-analyses did not show publication bias (*t* = 0.05, *p* = 0.96; *t* = 0.46, *p* = 0.67, respectively). The funnel plots can be found in Supplementary Figures 4, 5 of the supplemental documents.

## Discussion

The primary outcome of this meta-analysis revealed that apathy was associated with a greater than 2-fold increased risk of incident cognitive impairment. However, unlike the previous meta-analysis (9), our systematic review included cohort studies comprising cognitively intact community samples. Compared with previous studies, our study conducted the OR and HR analyses separately. Both analyses generated similar results and thus confirmed the association between apathy and cognitive impairment. Besides, the subgroup analyses per sampling settings, the sex distribution, age, and ApoE genotyping status further consolidated the findings of our study. Therefore, it might extend the results in a more generalizable manner to the broader older population.

The results support the hypothesis that apathy is predominantly prodromal to cognitive impairment among those in the normal cognitive population. The predictive value was diminished with longer follow-up times. It is plausible to hypothesize that apathy-associated patterns of dementia-related atrophy start at the stage of normal cognitive function. Apathy has been closely associated with components of the frontostriatal circuit (19, 20). The frontostriatal circuit, linking the ventral striatum to the dorsal anterior cingulate cortex (ACC) via the ventral pallidum and thalamus, is crucially involved in effort-based decision making and executive functions (20). Apathy has been confirmed to be substantially associated with executive function deterioration (21). Apathy could also contribute to cognitive impairment by driving metabolic abnormalities (22). The Alzheimer's Disease Neuroimaging Initiative (ADNI) study demonstrated a correlation between posterior cingulate cortex (PCC) hypometabolism and higher apathy scores (23). Subgroups of studies showed that apathy has the highest impact among the oldest population. The older population is more often due to neurodegenerative processes, which provides more evidence for apathy more likely to be a prodromal syndrome in the CN population.

In this meta-analysis, apathy could predict both MCI and dementia but not cognitive decline as measured by MMSE scores, suggesting that apathy may be less helpful in predicting cognitive change below the MCI threshold. It is reasonable that higher predictions could be obtained in clinical samples than in general community-dwelling older populations, as clinical samples might have much more severe conditions than the general population. Regarding the other results of our subgroup analysis, there was not much difference based on ApoE adjustment, suggesting that apathy could independently predict cognitive impairment regardless of ApoE genetic status. A previous meta-analysis also showed no association between APOE carriership and the presence of apathy (24).

Apathy was hard to differentiate from depression in the cognitively normal population. Apathy is characterized by decreased salience-related processing in the anterior cingulate cortex, whereas depression is characterized by increased salience-related processing (25). The two syndromes also differ on a neurochemical basis. Apathy may be associated with cholinergic deficits. However, depression may be associated with serotoninergic deficits or a dopamine-norepinephrine imbalance (26). In our study, we did not find an association after depression adjustment. However, the depression adjustment subgroup contained only two articles, and both used a depression-specific scale, which might lack sensitivity for detecting apathy. Besides, apathy is not easy to evaluate or detect, as it has three different domains. The variability of symptoms across domains may complicate the assessment. Most studies used NPI or scale of depression to evaluate neuropsychiatric syndromes, which are not specific for detecting apathy. Therefore, in future research, more work is warranted to confirm the association of apathy and cognitive impairment after adjusting for depressive symptoms and using more sensitive apathy measurements with specific tools, such as apathy evaluation scaly (AES) or Apathy Motivation Index in the general population (27).

## Limitations

Our review has some limitations. First, the value of apathy in predicting cognitive impairment development was verified, but the heterogeneity was high. We thus performed subgroup analyses to try to identify sources of heterogeneity. Second, subgroup analyses explained how some characteristics influenced the association between apathy and cognitive impairment, but the number of studies in each subgroup, such as depression adjustment status, was limited. Therefore, the results should be cautiously interpreted. Third, most studies in this meta-analysis used the NPI or GDS. However, these are validated scales; they are not specifically designed for measuring apathy, as is the apathy evaluation scale, which might have diluted the associations. Last, our study only conducted a comprehensive search of the major electronic English databases but did not include unpublished data, gray literature, and those published in other languages. Thus, the likelihood of a publication bias might exist. Further studies should consider these factors.

## Conclusions

In conclusion, this meta-analysis adds to previous evidence regarding apathy as a significant risky mental state for MCI and dementia for those in the general population. The findings support the concept of mild behavioral impairment as a prodromal syndrome to dementia (28). Older people with apathy were less likely to engage in social activities and were less motivated to seek clinicians' assistance; they were very vulnerable to cognitive impairment. In future research, more sensitive measurements are needed to detect apathy symptoms in the general population. In addition, more research is needed to clarify whether the association between apathy and conversion to cognitive impairment changed with adjustments for confounding variables.

## Author Contributions

ZF and LW contributed to the study extraction, data analysis, and drafted the manuscript. XL and HZ made some comments. CY, LT, YZ, and MZ provided some suggestions on how to revise the paper. XY and HW contributed to the design of this study, interpretation of data, and critical revision of the manuscript. All authors contributed to manuscript revision, read, and approved the submitted version.

## Funding

This work was funded by China's National Key R&D Program (XY, Grant Number: 2018YFC1314200; HW, Grant Number: 2017YFC1311100).

## Conflict of Interest

The authors declare that the research was conducted in the absence of any commercial or financial relationships that could be construed as a potential conflict of interest.

## Publisher's Note

All claims expressed in this article are solely those of the authors and do not necessarily represent those of their affiliated organizations, or those of the publisher, the editors and the reviewers. Any product that may be evaluated in this article, or claim that may be made by its manufacturer, is not guaranteed or endorsed by the publisher.

## References

[1] Krell-Roesch J Syrjanen JA Mielke MM Christianson TJ Kremers WK Machulda MM . Association between neuropsychiatric symptoms and functional change in older non-demented adults: mayo clinic study of aging. J Alzheimers Dis. (2020) 78:911–7. 10.3233/JAD-200764 33074231 PMC7794056

[2] Brodaty H Heffernan M Draper B Reppermund S Kochan NA Slavin MJ . Neuropsychiatric symptoms in older people with and without cognitive impairment. J Alzheimers Dis. (2012) 31:411–20. 10.3233/JAD-2012-120169 22571979

[3] Liew TM. Symptom clusters of neuropsychiatric symptoms in mild cognitive impairment and their comparative risks of dementia: a cohort study of 8530 older persons. J Am Med Dir Assoc. (2019) 20:1054. 10.1016/j.jamda.2019.02.012 30926409 PMC6663577

[4] Sperling RA Aisen PS Beckett LA Bennett DA Craft S Fagan AM . Toward defining the preclinical stages of Alzheimer's disease: recommendations from the national institute on aging-alzheimer's association workgroups on diagnostic guidelines for alzheimer's disease. Alzheimers Dement. (2011) 7:280–92. 10.1016/j.jalz.2011.03.003 21514248 PMC3220946

[5] Geda YE Roberts RO Knopman DS Petersen RC Christianson TJH Pankratz VS . Prevalence of neuropsychiatric symptoms in mild cognitive impairment and normal cognitive aging. Arch Gen Psychiatry. (2008) 65:1193–8. 10.1001/archpsyc.65.10.1193 18838636 PMC2575648

[6] Robert P Lanctôt KL Agüera-Ortiz L Aalten P Bremond F Defrancesco M . Is it time to revise the diagnostic criteria for apathy in brain disorders? the 2018 international consensus group. Eur Psychiatry. (2018) 54:71–6. 10.1016/j.eurpsy.2018.07.008 30125783

[7] Huang S-S Lee M-C Liao Y-C Wang W-F Lai T-J. Caregiver burden associated with behavioral and psychological symptoms of dementia (BPSD) in Taiwanese elderly. Arch Gerontol Geriatr. (2012) 55:55–9. 10.1016/j.archger.2011.04.009 21601931

[8] Saari T Hallikainen I Hintsa T Koivisto AM. Neuropsychiatric symptoms and activities of daily living in Alzheimer's disease: ALSOVA 5-year follow-up study. Int Psychogeriatrics. (2020) 32:741–51. 10.1017/S1041610219001571 31656211

[9] van Dalen JW van Wanrooij LL Moll van Charante EP Brayne C van Gool WA Richard E. Association of apathy with risk of incident dementia. JAMA Psychiatry. (2018) 75:1012. 10.1001/jamapsychiatry.2018.1877 30027214 PMC6233800

[10] Acosta I Borges G Aguirre-Hernandez R Sosa AL Prince M. Neuropsychiatric symptoms as risk factors of dementia in a Mexican population: a 10/66 dementia research group study. Alzheimers Dement. (2018) 14:271–9. 10.1016/j.jalz.2017.08.015 29028481 PMC5869051

[11] Ceïde ME Warhit A Ayers EI Kennedy G Verghese J. Apathy and the risk of predementia syndromes in community-dwelling older adults. J Gerontol Ser B. (2020) 75:1443–50. 10.1093/geronb/gbaa063 32374839 PMC7424283

[12] Geda YE Roberts RO Mielke MM Knopman DS Christianson TJH Pankratz VS . Baseline neuropsychiatric symptoms and the risk of incident mild cognitive impairment: a population-based study. Am J Psychiatry. (2014) 171:572–81. 10.1176/appi.ajp.2014.13060821 24700290 PMC4057095

[13] Wells G Shea B O'Connell D Peterson J Welch V Losos M. The Newcastle-Ottawa Scale (NOS) For Assessing The Quality If Nonrandomized Studies In Meta-Analyses. (2012). Available online at: http//www.ohri.ca/programs/clinical_epidemiology/oxford.asp

[14] van der Linde RM Stephan BCM Matthews FE Brayne C Savva GM. The presence of behavioural and psychological symptoms and progression to dementia in the cognitively impaired older population. Int J Geriatr Psychiatry. (2013) 28:700–9. 10.1002/gps.3873 22887592

[15] Clarke DE Ko JY Lyketsos C Rebok GW Eaton WW. Apathy and cognitive and functional decline in community-dwelling older adults: results from the Baltimore ECA longitudinal study. Int Psychogeriatrics. (2010) 22:819–29. 10.1017/S1041610209991402 20478091 PMC2893259

[16] Liew TM. Neuropsychiatric symptoms in cognitively normal older persons, and the association with Alzheimer's and non-Alzheimer's dementia. Alzheimers Res Ther. (2020) 12:35. 10.1186/s13195-020-00604-7 32234066 PMC7110750

[17] Burke SL Maramaldi P Cadet T Kukull W. Neuropsychiatric symptoms and Apolipoprotein E: Associations with eventual Alzheimer's disease development. Arch Gerontol Geriatr. (2016) 65:231–8. 10.1016/j.archger.2016.04.006 27111252 PMC5029123

[18] Masters MC Morris JC Roe CM. “Noncognitive” symptoms of early Alzheimer disease: a longitudinal analysis. Neurology. (2015) 84:617–22. 10.1212/WNL.0000000000001238 25589671 PMC4335988

[19] Le Heron C Apps MAJ Husain M. The anatomy of apathy: a neurocognitive framework for amotivated behaviour. Neuropsychologia. (2018) 118:54–67. 10.1016/j.neuropsychologia.2017.07.003 28689673 PMC6200857

[20] Nobis L Husain M. Apathy in Alzheimer's disease. Curr Opin Behav Sci. (2018) 22:7–13. 10.1016/j.cobeha.2017.12.007 30123816 PMC6095925

[21] Kawagoe T Onoda K Yamaguchi S. Apathy and executive function in healthy elderly—resting state fMRI study. Front Aging Neurosci. (2017) 9:124. 10.3389/fnagi.2017.00124 28536519 PMC5422524

[22] Ng KP Chiew HJ Rosa-Neto P Kandiah N Ismail Z Gauthier S. Brain metabolic dysfunction in early neuropsychiatric symptoms of dementia. Front Pharmacol. (2019) 10:1–8. 10.3389/fphar.2019.01398 31824321 PMC6882863

[23] Gatchel JR Donovan NJ Locascio JJ Becker JA Rentz DM Sperling RA . Regional 18F-fluorodeoxyglucose hypometabolism is associated with higher apathy scores over time in early alzheimer disease. Am J Geriatr Psychiatry. (2017) 25:683–93. 10.1016/j.jagp.2016.12.017 28410856 PMC5906700

[24] Banning LCP Ramakers IHGB Deckers K Verhey FRJ Aalten P. Apolipoprotein E and affective symptoms in mild cognitive impairment and Alzheimer's disease dementia: a systematic review and meta-analysis. Neurosci Biobehav Rev. (2019) 96:302–15. 10.1016/j.neubiorev.2018.11.020 30513312

[25] Onoda K Yamaguchi S. Dissociative contributions of the anterior cingulate cortex to apathy and depression: topological evidence from resting-state functional MRI. Neuropsychologia. (2015) 77:10–8. 10.1016/j.neuropsychologia.2015.07.030 26235668

[26] Mortby ME Maercker A Forstmeier S. Apathy: a separate syndrome from depression in dementia? a critical review. Aging Clin Exp Res. (2012) 24:305–16. 10.3275/8105 22102508

[27] Klar VS Ang Y Lockwood P Attaallah B Dickson S Drew D . Assessment of apathy in neurological patients using the apathy motivation index caregiver version. J Neuropsychol. (2021). 10.1111/jnp.12262. [Epub ahead of print]. 34532963 PMC9290131

[28] Creese B Brooker H Ismail Z Wesnes KA Hampshire A Khan Z . Mild behavioral impairment as a marker of cognitive decline in cognitively normal older adults. Am J Geriatr Psychiatry. (2019) 27:823–34. 10.1016/j.jagp.2019.01.215 30902566

