# Supplementary material for: Apathy as a Risky Neuropsychiatric Syndrome of Progression From Normal Aging to Mild Cognitive Impairment and Dementia: A Systematic Review and Meta-Analysis
Source: Front Psychiatry. 2021 Dec 20;12:792168. doi: 10.3389/fpsyt.2021.792168 (PMC8721876; doi:10.3389/fpsyt.2021.792168)
Supplement: Supplementary file 2 [file Data_Sheet_1.docx]

Searching term (PubMed as example)

**Pubmed:**

("Observational Studies as Topic"[Mesh] OR "Observational Study" [Publication Type] OR "cohort studies"[MeSH] OR "cohort"[Title/Abstract] OR "risk"[Title/Abstract] OR "Prospective"[Title/Abstract] OR "follow up"[Title/Abstract]) AND ((“dementia”[MESH] OR “dementia”[Title/Abstract] OR "cognitive impairment" [Title/Abstract] OR "Cognitive Decline"[Title/Abstract] OR "Alzheimer disease" [Title/Abstract]) AND ((apathy[Title/Abstract]) OR (Motivation[Title/Abstract])) OR (neuropsychiatric symptoms[Title/Abstract])) OR ("Apathy"[Mesh]))


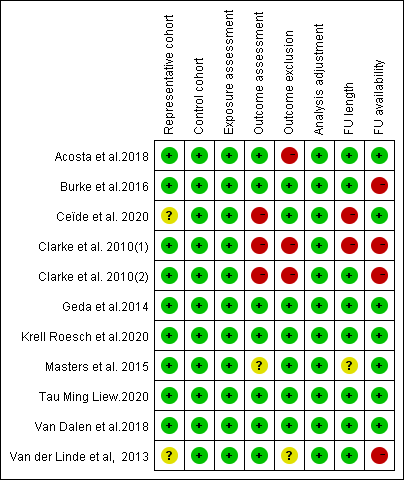


Figure S1. Risk of bias table

For each category, green color indicates low risk of bias, yellow color indicates intermediate risk of bias, and red color indicates high risk of bias or insufficient information available on the subject. For most categories, green and yellow color scores 1 point, red color no points. For“Analysis adjustment”green color scores 2 points (1 point. Study controls for age and sex; 1 point. Study controls for any additional factor: education, depression, ApoE and cognitive function). FU: follow-up


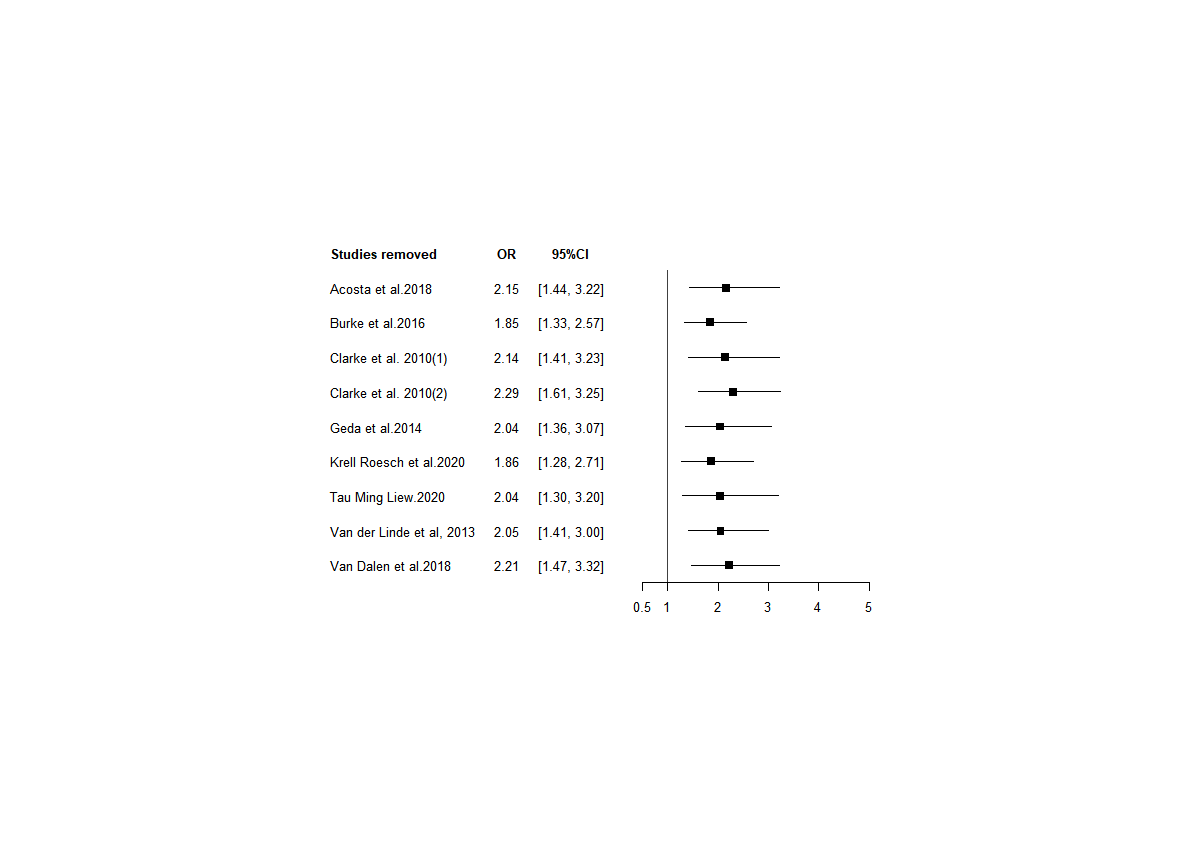


Figure S2. Sensitivity plot for OR meta-analysis.


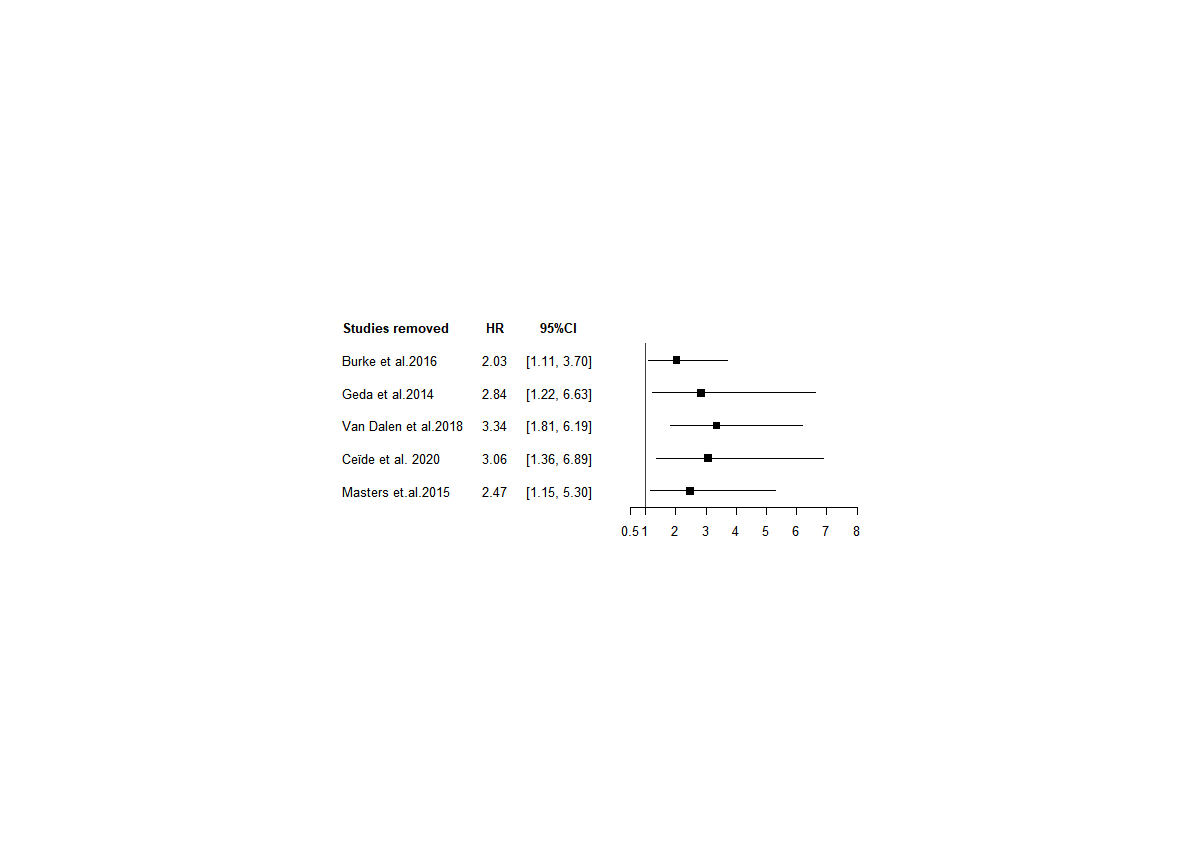


Figure S3. Sensitivity plot for HR meta-analysis


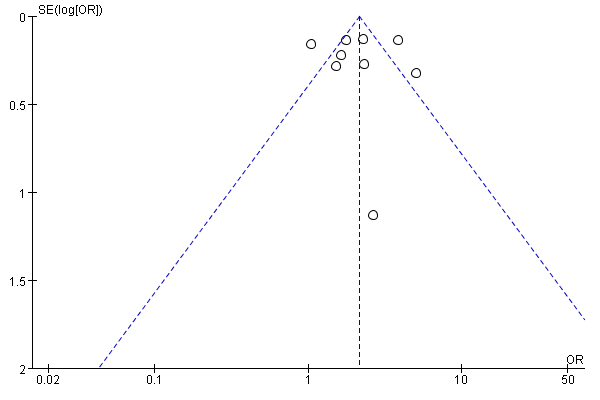


Figure S4. Funnel plot of risk ratios reported in studies for OR meta-analysis


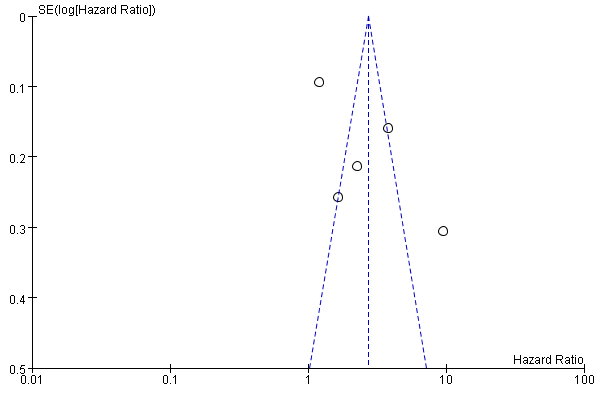


Figure S5. Funnel plot of risk ratios reported in studies for HR meta-analysis
